# Evaluation of Analytical and Clinical Performance and Usefulness in a Real-Life Hospital Setting of Two in-House Real-Time RT-PCR Assays to Track SARS-CoV-2 Variants of Concern

**DOI:** 10.3390/v15051115

**Published:** 2023-05-04

**Authors:** Alice Moisan, Anaïs Soares, Fabienne De Oliveira, Elodie Alessandri-Gradt, François Lecoquierre, Steeve Fourneaux, Jean-Christophe Plantier, Marie Gueudin

**Affiliations:** 1 Univ Rouen Normandie, UNICAEN, INSERM, DYNAMICURE UMR 1311, CHU Rouen, Department of Virology, F-76000 Rouen, France; 2 Department of Genetics and Reference Center for Developmental Disorders, FHU G4 Génomique, Normandie University, UNIROUEN, CHU Rouen, INSERM U1245, 76000 Rouen, France

**Keywords:** variants of concern, SARS-CoV-2 diversity, in-house RT-PCR, next-generation sequencing, mutations of interest

## Abstract

Since the end of 2020, multiple severe acute respiratory syndrome coronavirus 2 (SARS-CoV-2) variants of concern (VOCs) have emerged and spread worldwide. Tracking their evolution has been a challenge due to the huge number of positive samples and limited capacities of whole-genome sequencing. Two in-house variant-screening RT-PCR assays were successively designed in our laboratory in order to detect specific known mutations in the spike region and to rapidly detect successively emerging VOCs. The first one (RT-PCR#1) targeted the 69–70 deletion and the N501Y substitution simultaneously, whereas the second one (RT-PCR#2) targeted the E484K, E484Q, and L452R substitutions simultaneously. To evaluate the analytical performance of these two RT-PCRs, 90 negative and 30 positive thawed nasopharyngeal swabs were retrospectively analyzed, and no discordant results were observed. Concerning the sensitivity, for RT-PCR#1, serial dilutions of the WHO international standard SARS-CoV-2 RNA, corresponding to the genome of an Alpha variant, were all detected up to 500 IU/mL. For RT-PCR#2, dilutions of a sample harboring the E484K substitution and of a sample harboring the L452R and E484Q substitutions were all detected up to 1000 IU/mL and 2000 IU/mL, respectively. To evaluate the performance in a real-life hospital setting, 1308 and 915 profiles of mutations, obtained with RT-PCR#1 and RT-PCR#2, respectively, were prospectively compared to next-generation sequencing (NGS) data. The two RT-PCR assays showed an excellent concordance with the NGS data, with 99.8% for RT-PCR#1 and 99.2% for RT-PCR#2. Finally, for each mutation targeted, the clinical sensitivity, the clinical specificity and the positive and negative predictive values showed excellent clinical performance. Since the beginning of the SARS-CoV-2 pandemic, the emergence of variants—impacting the disease’s severity and the efficacy of vaccines and therapies—has forced medical analysis laboratories to constantly adapt to the strong demand for screening them. Our data showed that in-house RT-PCRs are useful and adaptable tools for monitoring such rapid evolution and spread of SARS-CoV-2 VOCs.

## 1. Introduction

**Figure 1:**
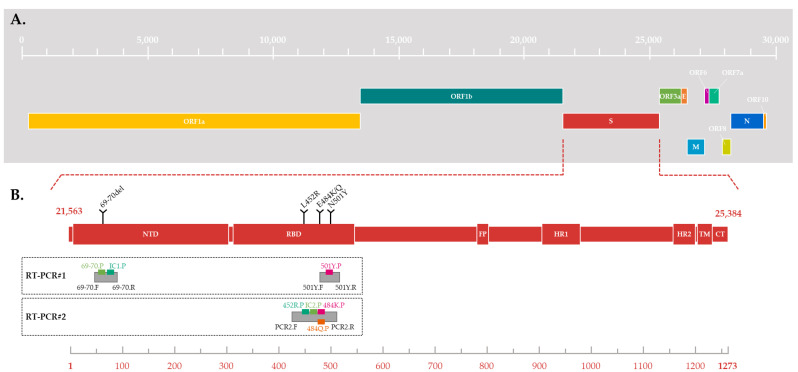
(**A**) Schematic of SARS-CoV-2 genome structure. The genes are located relative to the numbering of the Wuhan-Hu 1 SARS-CoV-2 reference strain (Genbank accession number MN908947.3). (**B**) Schematic of SARS-CoV-2 spike protein structure. Different domains are shown: NTD = N-terminal domain, RBD = receptor-binding domain; FP = fusion peptide, HR1 = heptad repeat 1, HR2 = heptad repeat 2, TM = transmembrane domain and CT = cytoplasmic tail (derived from [7,8]). The substitutions targeted by the two RT-PCR are located relative to the spike aminoacid numbering. The location of the amplicon is represented by grey boxes, with the hybridization location of probes and primers for each RT-PCR.

The unprecedented COVID-19 pandemic was caused by the severe acute respiratory syndrome coronavirus 2 (SARS-CoV-2), carrying one of the largest viral RNA genomes and encoding about 29 proteins, including 4 structural proteins (spike (S), envelope (E), membrane (M), and nucleocapsid (N)) [1,2,3,4,5,6] (Figure 1A).

Since the end of 2020, five SARS-CoV-2 variants with several mutations of interest in S glycoprotein have rapidly spread worldwide and are associated with an increased risk to global public health. These variants were characterized as variants of concern (VOCs) by the World Health Organization (WHO), because of the presence of mutations in the S glycoprotein (including the 69–70 deletion in the N-terminal domain and the N501Y, E484K/Q, and L452R substitutions in the receptor binding domain (RDB) (Figure 1B)), and their impact on the associated disease’s severity, the efficacy of vaccines and therapies, the performance of diagnostic tools, or other public health and social measures [9,10,11,12,13,14,15,16,17,18,19,20,21,22,23,24]. A nonstigmatizing nomenclature using letters of the Greek alphabet was also adopted for these five VOCs: Alpha [25], Beta [26], Gamma [27], Delta [28], and Omicron [29]. The large-scale monitoring of these variants’ evolution in a population and in a territory is a challenge which requires sequencing a large number of SARS-CoV-2 whole genomes from positive samples. Due to the huge volume of positive samples and the limited capacities of sequencing, a selection must be made before sequencing. In January 2021, when the Alpha, Beta, and Gamma VOCs began to circulate in France, the French government adopted a strategy to curb their spread based on their early detection and the strengthening of contact tracing [30]. Thus, at the end of January 2021, in order to quickly detect an infection by a VOC, a variant-screening reverse transcription-PCR (RT-PCR) assay based on specifically targeting several known mutations became mandatory for all SARS-CoV-2-positive samples [30]. This systematic search for one or more nucleotide substitutions using real-time PCR has a significant advantage over random sequencing-based surveillance strategies in terms of time, cost, and adaptability.

**Figure 2:**
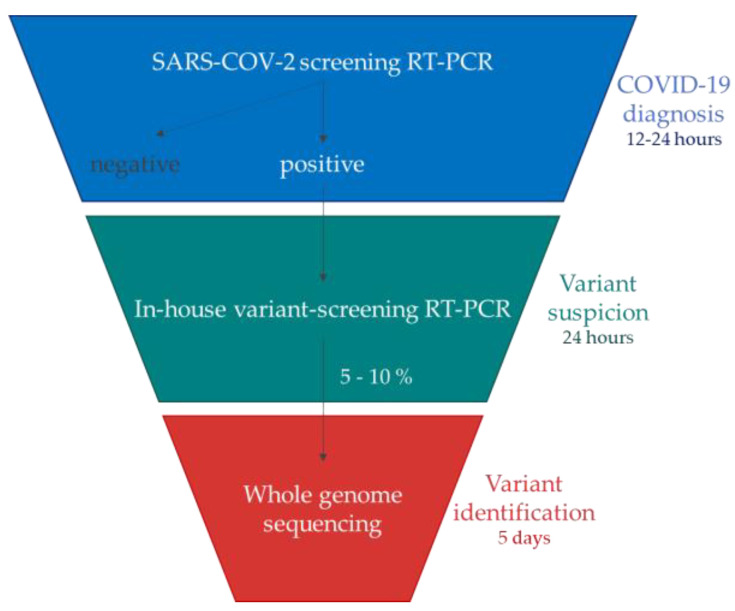
Algorithm used to characterize and rapidly detect SARS-CoV-2 VOCs which have successively emerged (derived from the French guidelines [30,31]).

In accordance with these French guidelines, the implementation of an RT-PCR-based algorithm to detect specific known mutations in the Spike region in all positive samples enabled the rapid detection of successively emerging VOCs within 24 h of a COVID-19 diagnosis [30,31] (Figure 2). For this, two in-house one-step variant-screening real-time RT-PCR assays were successively designed in our laboratory to detect the variants but also to select the strains to undergo next-generation sequencing (NGS).

**Figure 3:**
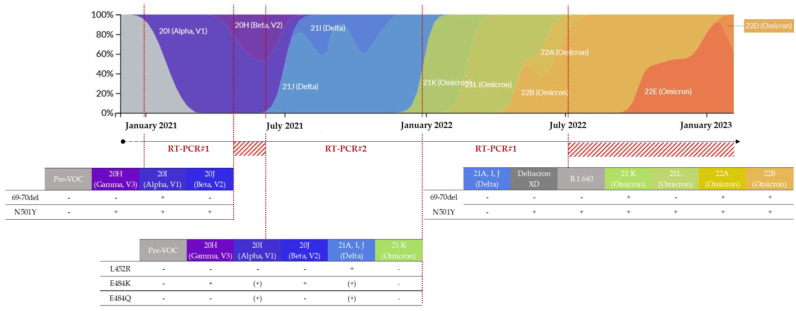
Frequencies of detection of the SARS-CoV-2 variants in France (colored by clade and normalized to 100% at each time point) [22] and evolution of the strategy of variant screening in our laboratory.

In practice, the first RT-PCR#1, used from February to May 2021, simultaneously targeted the 69–70 deletion and the N501Y substitution (Figure 1B and Figure 3). Depending on the combinations of detected mutations, a suspicion of infection by the Alpha VOC could be differentiated from that of the Beta or Gamma VOC and from that of a pre-VOC “wild” variant. The second RT-PCR#2, used from June to December 2021, simultaneously targeted three substitutions: E484K, E484Q, and L452R (Figure 1B). The different combinations of these substitutions enabled discriminating numerous variants, including circulating VOCs. During late 2021, RT-PCR#2 was replaced by RT-PCR#1 due to the circulation and diffusion of the Omicron variant in France (Figure 3) [32]. In July 2022, the viral diversification of the Omicron wave led us to change the targeted mutations again.

RT-PCR#1 was used from February to May 2021, then from December 2021 to July 2022. The RT-PCR#2 was used from June to December 2021. The red hashed areas correspond to a period of using other combinations of substitutions. The presence and the absence of the mutation are represented by + and −, respectively. Additionally, (+) corresponds to the inconstant presence of the mutation in the clade/lineage.

The aim of this work was to evaluate the analytical and clinical performance of our two in-house variant-screening real-time RT-PCR assays to track SARS-CoV-2 variants of concern according to the “STARD 2015” checklist [33].

## 2. Materials and Methods

### 2.1. Clinical Samples and International Standard

The analytical and clinical performance of the two variant-screening RT-PCR assays were evaluated from nasopharyngeal swabs with viral transport medium, collected between 27 January 2021 and 28 February 2022, and referred to the laboratory for SARS-CoV-2 testing. On arrival, the samples were tested with the RealStar^®^ SARS-CoV-2 RT-PCR Kit 1.0 (Altona Diagnostic, Hamburg, Germany) [34].

For the evaluation of the analytical performance, 90 SARS-CoV-2 RNA-negative samples were selected, thawed, and retrospectively analyzed with each variant-screening RT-PCR. Additionally, 60 positive samples (30 for RT-PCR#1 and 30 for RT-PCR#2), sequenced with NGS and representing the circulating variability in SARS-CoV-2 in France, were retrospectively selected and thawed to verify the correspondence between the mutations observed with each variant-screening RT-PCR and the consensus sequence generated with NGS. Then, all the strains screened by one of the two RT-PCRs and sequenced with NGS were prospectively included in evaluating the clinical performance and the performance in a real-life hospital setting. Thus, the results obtained from 2221 (1308 and 915 for RT-PCR#1 and #2, respectively) SARS-CoV-2-positive samples collected between February 2021 and February 2022 were analyzed as the samples arrived.

To determine linearity, efficiency, and analytical sensitivity, the first WHO International Standard for SARS-CoV-2 RNA (WHO-IS SCV-2 RNA) was used. This standard was designed for nucleic-acid-amplification-based assays and consists of the acid–heat-inactivated England/02/2020 isolate of SARS-CoV-2 (National Institute for Biological Standards and Control, London, UK). This isolate notably harbors the 69–70 deletion and the N501Y substitution in its Spike region. After reconstitution, according to the supplier’s instructions, the final viral load was 7.7 Log_10_ IU/mL.

### 2.2. RNA Extraction

First, each sample was heat-inactivated with incubation at 56 °C for 30 min and 180 µL was dispatched in a 96-well plate. For RNA extraction, the MGIEasy Magnetic Beads Virus DNA/RNA Extraction Kit was used on MGI SP-960 instruments (MGI Tech Co., Shenzhen, China) according to the manufacturer’s recommendations.

### 2.3. SARS-CoV-2 Variant-Screening RT-PCR Assays

RT-PCR#1 was based on a multiplex system, using two pairs of primers and three probes to target the S gene (Table 1). A pair of primers and two probes were designed to detect a specific but conserved sequence of SARS-CoV-2 and the 69–70 deletion (Figure 1B). The first target was associated with the probe labelled with the fluorophore FAM and served as an internal control (IC) to check that the RT-PCR worked properly for each sample.

Thus, the FAM signal should be detected when a SARS-CoV-2 virus is present in the sample, whatever the variant. The deletion was pointed out with the probe labelled with the fluorophore JOE: the JOE signal should be detected when the deletion is absent. A second pair of primers and a probe targeted the N501Y substitution (Figure 1B), which was associated with the probe labelled with the fluorophore Cy5. The Cy5 signal should be detected when the mutation is absent.

In the same way, RT-PCR#2 was based on a multiplex system but used a single pair of primers and four probes to target four distinct sequences of the S gene simultaneously (Table 1). This combination of probes should enable the detection of a specific but conserved sequence of SARS-CoV-2, serving as an IC, and of three S-gene mutations of interest: E484K, E484Q, and L452R (Figure 1B). The SARS-CoV-2 target, the E484K, the E484Q, and the L452R substitutions, were associated with the probes labelled with the fluorophores JOE, Cy5, ROX, and FAM, respectively. Unlike RT-PCR#1, a signal should be detected when the mutation is present.

All oligonucleotides were designed with Oligo (Primer analysis software v6.41) after selecting the targeted regions of the SARS-CoV-2 genome, based on an alignment of sequences representative of viral diversity performed with MEGA7 [35]. All the primers and probes were synthetized by Eurogentec (Seraing, Belgium) and used at a concentration of 10 µM.

A 20 μL RT-PCR reaction mix was performed using the Superscript III Platinum One-step Quantitative RT-PCR kit (Invitrogen life technologies, Carlsbad, CA, USA); each RT-PCR tube contained 12.5 μL of 2X reaction mix (containing 0.4 mM of each deoxyribonucleotide triphosphate (dNTP) and 6 mM magnesium sulfate), 1 μL of reverse transcriptase/Taq mixture, 0.4 μL of a 50 nM magnesium sulfate solution from the kit, 2.75 μL of primers and probes mix and 3.35 μL of water for RT-PCR#1, and 2 μL of primers and probes mix and 4.1 μL of water for RT-PCR#2. The primer and probe sequences and the optimized volumes are shown in Table 1. A volume of 10 μL of RNA was added to the RT-PCR reaction mix. Positive and negative controls were included on each plate. Thermal cycling was performed at 55 °C for 20 min for reverse transcription, followed by 95 °C for 3 min, and then 50 cycles of 95 °C for 15 s, and 58 °C for 30 s. RT-PCR and signal interpretation were performed on CFX96™ Real-Time PCR Detection System (Bio-Rad) instruments.

### 2.4. Data Analysis

The RT-PCR raw data produced by the CFX96 were processed through in-house python-based software (ACORS—Automated Classification Of RT-PCR Results for SARS-CoV-2), whose objectives were to automate the classification of RT-PCR profiles and to format and export the results to the LIMS (Laboratory Information Management System).

Briefly, a graphical interface allowed the user to import the raw data (.csv) and perform either a de novo screening or a variant-screening analysis. Fluorescence data were processed to compute the Ct (cycle threshold) via linear regression, evaluate the signal of each target, and, therefore, deduce the mutational profile and the potential variant. The RT-PCR curves are classified into three categories (positive, negative, and suspicious) using three parameters for which thresholds have been set: (i) the maximum fluorescence, (ii) the maximum slope of the exponential stage (maximum fluorescence delta between two cycles), and (iii) the computed Ct. The RT-PCR amplification profiles and classifications were displayed in the interface for user approval before formatting, export, and PDF report.

At the end of the process, the final analysis was qualitative and identified the presence or the absence of each targeted mutation. During the interpretation of a run, the absence of a Ct value was consistent with the absence of the targeted mutation, whereas the presence of a Ct value was consistent with its presence. Thresholds were set within the exponential phase of PCR and were fixed to maintain consistency in Ct value calculations each time the assay was run.

### 2.5. Analytical Performance of SARS-CoV-2 Variant-Screening RT-PCR Assays

The evaluation of analytical performance included assessing analytical sensitivity and specificity, linearity, and efficiency.

To evaluate the analytical sensitivity (i.e., the limit of detection) of RT-PCR#1, WHO-IS SCV-2 RNA, corresponding to the genome of an Alpha variant harboring the 69–70 deletion but not the N501Y substitution, was used. Serial dilutions were performed in viral transport medium to obtain three aliquots at 1000, 500, and 250 IU/mL. Ten replicates of each dilution were independently tested by RT-PCR#1. For RT-PCR#2, the WHO-IS SCV-2 RNA was only positive for the IC target which amplified all the SARS-CoV-2 variants. This property was used to quantify two positive samples previously sequenced with NGS with a range of WHO-IS SCV-2 RNA. The first one harbored the E484K substitution whereas the second one harbored the L452R and E484Q substitutions. After their quantification, both were serially diluted to obtain three aliquots at 2000, 1000, and 500 IU/mL. Ten replicates of each dilution were independently tested with RT-PCR#2.

To assess analytical specificity, the percentage of negative results obtained among the 90 retrospectively analyzed SARS-CoV-2 RNA-negative samples was calculated for each RT-PCR.

Then, the linearity and efficiency of the amplification of both RT-PCRs were evaluated. For this, the WHO-IS SCV-2 RNA was serially diluted in viral transport medium with five 10-fold dilutions from 6.7 to 2.7 Log_10_ IU/mL and three 2-fold dilutions from 2.4 to 1.8 Log_10_ IU/mL. All of these 8 serial dilutions were amplified, each in duplicate.

RT-PCR#1 and RT-PCR#2 were intended as reflex assays following primary PCR detection of SARS-CoV-2, and therefore, specificity to other respiratory viruses was not considered in our evaluations.

### 2.6. Validation in a Real-Life Hospital Setting of SARS-CoV-2 Variant-Screening RT-PCR Assays

In order to validate the PCR performance and detect potential discordances, the results obtained from 2221 samples collected between February 2021 and February 2022 were analyzed. The results from RT-PCR#1 (n = 1308), RT-PCR#2 (n = 915), or RT-PCR#1+RT-PCR#2 (n = 5) were compared to the variant identified with sequencing and the percent agreement, corresponding to the proportion of similar results between the variant-screening assay and NGS, was calculated for each RT-PCR.

Whole genomes were sequenced with NGS on samples with a variant-screening RT-PCR Ct of less than 30, using an Oxford Nanopore Technologies MinION Mk1C device (ONT, Oxford, UK). Sequencing libraries were prepared according to the ARTIC LoCost V.3 protocol [36], which consists of an amplicon-based approach using V3, V4, then V4.1 primers (IDT, Coralville, IA, USA). The final library was sequenced for 24 h.

SARS-CoV-2 consensus genomes were generated from February to April 2021, according to the nCoV-2019 novel coronavirus bioinformatics protocol developed by the Connor Lab [37], and from April 2021, according to an in-house pipeline derived from Epi2me Labs workflow (ONT). The clades (component mutations) [38] and the lineages (phylogenetic distribution) [39] were assigned after Nextclade and Pango lineage analysis [40,41,42] and all data were submitted to the EMERGEN and GISAID databases [43,44].

### 2.7. Clinical Performance of SARS-CoV-2 Variant-Screening RT-PCR Assays

The evaluation of clinical performance included assessing clinical sensitivity, specificity, positive predictive value (PPV) and negative predictive value (NPV). For this, the number of true presence, true absence, false presence, and false absence of each targeted mutation of each RT-PCR assay was determined, using the NGS results as a gold standard. Indeed, in this study, the presence and the absence of a targeted mutation were considered positive and negative results, respectively.

## 3. Results

### 3.1. Analytical Performance of SARS-CoV-2 Variant-Screening RT-PCR Assays

RT-PCR#1: In total, 30 SARS-CoV-2 positive and 90 SARS-CoV-2 negative nasopharyngeal swabs analyzed with variant-screening RT-PCR#1 were positive and negative, respectively, corresponding to an analytical specificity of 100.0% (Table 2).

During the first period of using RT-PCR#1, from February to May 2021 (n = 619), the association between the 69–70 deletion and N501Y substitution was consistent with an Alpha variant, the predominant variant at this time, whereas an isolated N501Y substitution suggested a Beta or Gamma variant (Figure 3, Table 3). During the second period of use, from December 2021 to February 2022 (n = 694), the absence of the 69–70 deletion and N501Y substitution suggested a Delta variant, whereas their presence implied a 21K Omicron (BA.1) variant. An isolated N501Y substitution could not discriminate a 21L Omicron (BA.2), or a Deltacron XD recombinant strain or B.1.640 strain, which were both involved in locally identified chains of transmission during this period [45,46] (Figure 3, Table 3).

Among the 30 positive samples, 13 harbored both the 69–70 deletion and the N501Y substitution, 15 harbored only the N501Y substitution, and 2 had neither. All 30 of these samples were fully sequenced with NGS. The distribution of clades/lineages was as follows: 20I (Alpha, V1) (n = 7, 23.3%), 20H (Beta, V2) (n = 7, 23.3%), 21K (Omicron, BA.1) (n = 6, 20.0%), 21L (Omicron, BA.2) (n = 5, 16.7%), 20J (Gamma, V3) (n = 3, 10.0%), and 21J (Delta) (n = 2, 6.7%). No discordance between the variant-screening RT-PCR#1 profile and whole-genome sequencing was observed, proving an excellent ability to predict the correct variant (Table 4).

**Figure 4:**
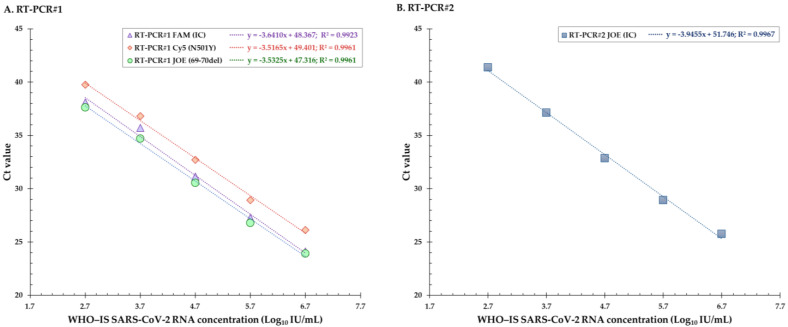
(**A**) Linear regression curves for the evaluation of linearity for the three fluorophores FAM, Cy5, and JOE of the RT-PCR#1. (**B**) Linear regression curve for the evaluation of linearity for the fluorophore JOE of the RT-PCR#2. Ct = cycle threshold, IC = internal control.

The 10 replicates of the WHO-IS SCV-2 RNA dilutions were all detected up to 500 IU/mL (2.7 Log_10_ IU/mL), with a mean Ct value of 36.9 for the FAM (IC) fluorophore. For the samples corresponding to 250 IU/mL, an amplification curve was observed for only 8 of the 10 dilutions. The linearity was obtained between 2.7 and 6.7 Log_10_ IU/mL, with a correlation coefficient (R^2^) between 0.9923 and 0.9961, depending on the fluorophore (Figure 4A). For the Cy5 fluorophore, both replicates and only one replicate diluted at 2.4 Log_10_ IU/mL and 2.1 Log_10_ IU/mL, respectively, were amplified, but none of the dilutions below 2.7 Log_10_ IU/mL were amplified for the FAM (IC) and JOE (69–70del) fluorophores (Table 5). The slope values of the linear regression curve were at 3.6410, 3.5165, and 3.5325, corresponding to an efficiency of 94.1, 96.2, and 96.0% for the FAM (IC), Cy5 (N501Y), and JOE (69–70del) fluorophores, respectively (Table 2, Figure 4A).

RT-PCR#2: In total, 30 SARS-CoV-2 positive and 90 SARS-CoV-2 negative nasopharyngeal swabs analyzed with variant-screening RT-PCR#1 were positive and negative, respectively, corresponding to an analytical specificity of 100.0% (Table 2). For all of them, the JOE signal, used as the internal control, was present. Due to the presence of three targets, several combinations of mutations could be observed, allowing discriminating numerous variants including circulating VOCs (Figure 3, Table 6). Thereby, an E484K+/E484Q−/L452R− profile was consistent with the Beta or Gamma variants, whereas an E484K−/E484Q−/L452R+ profile was consistent with the Delta variant, which was the new predominant variant during this period. The other combinations corresponded to variants of interest (VOIs), for which epidemiological surveillance surveys based on whole-genome sequencing (WGS) had shown a low level of circulation in France (Figure 3, Table 6).

Among the 30 positive samples, 8 and 10 strains harbored the L452R or the E484K substitutions, respectively, 9 strains showed an association between these substitutions, and 3 had neither of them. All 30 of these samples were fully sequenced with NGS. The distribution of clades/lineages was as follows: 21A/I/J (Delta) (n = 15, 50%), 20H (Beta, V2) (n = 5, 16.7%), 20I (Alpha, V1) (n = 2, 6.7%), 21K (Omicron, BA.1) (n = 2, 6.7%), 20J (Gamma, V3) (n = 1, 3.3%), and diverse VOIs (n = 5, 16.7%) (Table 4). No discordance between the variant-screening RT-PCR#2 profile and WGS was observed, proving an excellent ability to predict the correct variant (Table 4).

The 10 replicates of the dilutions of the sample presenting the E484K substitution were all detected up to 1000 IU/mL (3 Log_10_ IU/mL). For the samples corresponding to 500 IU/mL, amplification curves were observed for only 4 of the 10 dilutions. The 10 replicates of the dilutions of the sample presenting the L452R and E484Q substitutions were all detected up to 2000 IU/mL (3.3 Log_10_ IU/mL). For the samples corresponding to 1000 IU/mL, amplification curves were observed for only 8 of the 10 dilutions. The linearity was obtained between 2.7 and 6.7 Log_10_ IU/mL, with a correlation coefficient (R^2^) at 0.9967 (Figure 4B). None of the dilutions below 2.7 Log_10_ IU/mL were amplified (Table 5). The slope values of the linear regression curve were at 3.9455, corresponding to an efficiency of 90.0% (Figure 4B).

### 3.2. Validation in a Real-Life Hospital Setting of SARS-CoV-2 Variant-Screening RT-PCR Assays

RT-PCR#1: Among the 1308 samples which had available RT-PCR#1 and NGS results, 701 (53.9%) 69–70 deletions and 983 (75.1%) N501Y substitutions were detected (Table 2). Only three (0.2%) discordances were highlighted. The RT-PCR curves of all three of them appeared very late or presented unusual characteristics (a nonsigmoid curve or with a curve plateau lower than usual).

RT-PCR#2: Among the 915 samples which had available RT-PCR#2 and NGS results, 783 (85.7%) L452R, 14 (1.5%) E484K, and 14 (1.5%) E484Q substitutions were detected (Table 2). Only seven discordances (0.8%) were detected: five and two relating to the L452R and the E484K substitutions, respectively. All of them corresponded to unusual curves, as observed for RT-PCR#1.

### 3.3. Clinical Performance of SARS-CoV-2 Variant-Screening RT-PCR Assays

For the two RT-PCRs combined, the clinical sensitivity and specificity ranged from 92.9% (E484K) to 100.0% (N501Y and E484Q) and from 96.2% (L452R) to 100.0% (69–70del, N501Y and E484Q), respectively (Table 2). PPV was 100.0%, with the exception of the E484K (92.9%) and L452R (99.4%) substitutions, targeted by the RT-PCR#2. NPV ranged from 99.2% (L452R) to 100.0% (N501Y and E484Q), with a mean value of 99.7% (Table 2).

## 4. Discussion

The emergence and the spread of new SARS-CoV-2 variants are associated with a risk of increased transmissibility and severity, reduced effectiveness in diagnostic tools, and/or immune and vaccine escapes [9]. At the end of January 2021, following the injunctions from the French government, a variant-screening RT-PCR assay very quickly became mandatory [30,31]. The 69–70 deletion and the N501Y and E484K substitutions had to be screened for all SARS-CoV-2-positive samples in order to detect an Alpha, Beta, or Gamma variant which shared an N501Y substitution. In May 2021, when the Delta VOC emerged in India, the French government applied new recommendations including the screening of new mutations of interest: E484K, E484Q, and L452R [47].

The COVID-19 pandemic has forced medical analysis laboratories to adapt quickly to meet both the extremely high demand for PCR screening tests and government orders concerning the monitoring of SARS-CoV-2 variants. Each time a VOC arrived in France, there was a need to search for substitutions or deletions using RT-PCR, but commercial kits were not yet available or officially validated. Thus, the design and the development of in-house variant-screening RT-PCR allowed rapid adaptation to demand without a loss of time or reagents, which constitutes the major advantage of our strategy. Indeed, since 2020, many RT-PCR assays designed for targeting mutations of interest have been developed to improve the monitoring of viral evolution over time. The first developed techniques often used a revelation based on melt curve analysis [48,49]. These have quickly been abandoned because a high level of user expertise was needed for interpreting the results. Some RT-PCRs targeting a single mutation have also been developed [50,51]. This approach allows the detection of a specific variant, but quickly becomes obsolete due to evolutionary convergence leading to the emergence of the same mutation in genetically divergent variants’ genomes. To circumvent this, it is necessary to perform several RT-PCRs on the same sample, which is not suitable for the variant screening of a large number of positive samples. The approach we chose was a multiplex RT-PCR, revealed in Taqman using MGB probes for the detection of point mutations. It appears to be the best approach, combining rapid development, easy adaptation to the circulating diversity, and the rapid obtainment of the result. Other teams have chosen this approach to monitor the evolution of SARS-CoV-2 variants [52]. In addition, numerous sets of primers and probes are now available, most of which are described in this work. This allows us to be very reactive when new variants or lineages emerge or when a suspicious epidemiological situation must be investigated, such as investigating a B.1.640 variant cluster [46] or an extended transmission event of the Deltacron XD recombinant [45]. More recently, with the emergence of the Omicron 22A (Omicron BA.4), 22B (Omicron, BA.5), and 22C (Omicron, BA.2.12.2) variants, using a new, already designed and available combination of primers and probes allowed us to detect their presence from a variant-screening assay without waiting for the sequencing results (Figure 2).

These variant-screening RT-PCR assays can be used on respiratory samples found positive for SARS-CoV-2 using another technique. They showed an excellent concordance with the NGS data, with 99.8% for RT-PCR#1 and 99.2% for RT-PCR#2. However, they cannot be used as a first-line assay because of the lower sensitivity than the PCRs developed for diagnosis. In both our in-house variant-screening assays, a specific but conserved SARS-CoV-2 sequence was targeted in order to constitute an internal control, which validates the good progress of the reaction. For both RT-PCRs, the Cts obtained during the amplification of this IC were always lower than that of the targeted mutations, excluding any risk of misinterpreting the result.

Two different strategies were used to design these in-house PCR assays, each with their advantages and disadvantages. For RT-PCR#1, an amplification curve meant an absence of the targeted mutation. This strategy enabled us to detect several mutations in the same amino acid position because the probe did not bind, regardless of the nature of the mutation. However, this strategy made reading the results much less intuitive since the absence of a curve indicated the presence of a mutation on the viral genome. The design of RT-PCR#2 was more conventional because the probes targeted the desired mutations and a curve only appeared if the mutation was present.

In a laboratory that processes a huge volume of samples, the manual interpretation of multiplex real-time PCR assays can be tedious and prone to human error. In the case of screening PCR assays, test manufacturers rarely provide automatic interpretation software. Here, we developed a program entirely adapted to our practice, which allows the obtaining of a very readable automatic report. The parameterization of cycle thresholds, colors, acceptable slope values, and the main interpretations are accessible via the administrator profile and are very easy to change, which allows the biologist to adapt them according to the user’s needs.

The evolution of the virus led to a considerable accumulation of mutations and a classification that goes in the direction of Omicron subvariants, where it is more difficult to select critical places of variability while ensuring the test maintains the level of sensitivity and sensitivity. Of course, NGS remains the gold standard for identifying variants since the whole viral genome is sequenced and analyzed. This method enables the detection of unknown mutations or even variants not yet described. However, a major limitation of NGS technologies is the cost, which may be prohibitive for many diagnostic laboratories. Furthermore, the complexity of the NGS workflow and the requirement for sophisticated instrumentation and bioinformatics expertise may pose significant barriers to NGS access in many laboratories. Moreover, variant-screening RT-PCR is particularly adapted to large-scale screening, whereas NGS is more a tool for population-based surveillance. Indeed, during epidemic peaks, at the height of our activity, nearly 2700 samples were screened per week (4 × 96 samples a day), whereas only 192 of them were sequenced per week.

In conclusion, we designed and implemented two in-house variant-screening real-time RT-PCR assays with good analytical and clinical performance and excellent results in a real-life hospital setting. Such assays are useful and adaptable tools for monitoring the rapid evolution and spread of successively emerging SARS-CoV-2 VOCs but cannot be dissociated from the epidemiological monitoring of viral diversity with WGS.

## Figures and Tables

**Table 1:** Sequences of primers and probes and associated fluorophore/quencher used in RT-PCR #1 and #2.

**RT-PCR#1**				
Targeted mutations	Oligonucleotide		Fluorophore-Sequence (5′ > 3′)-Quencher	Volume for one tube of dilution at 10 µM
69–70 deletion	69–70.F	*Primer Forward 152U21*	CTCAGGACTTGTTCTTACCTT	0.5 µL
	69–70.R	*Primer Reverse 262L20*	GAAGCAAAATAAACACCATC	0.5 µL
	IC1.P	*Probe IC SARS-CoV-2*	FAM–TCTCTGGGACCAATGGTACT–BHQ1	0.25 µL
	69–70.P	*Probe 69–70 deletion*	JOE–TGGTCCCAGAGACATGTATAGC–BHQ1	0.25 µL
N501Y substitution	501Y.F	*Primer Forward N501Y*	GGTGTTRAAGGTTTTAATTGTTAC	0.5 µL
	501Y.R	*Primer Reverse N501Y*	TTTTAGGTCCACAAACAGTTGC	0.5 µL
	501Y.P	*Probe SARS-CoV-2 N501Y*	Cy5-CCAACCCACTAATGGTGT–MGB	0.25 µL
**RT-PCR#2**				
Targeted mutations	Oligonucleotide		Fluorophore-Sequence (5′ > 3′)-Quencher	Volume for one tube of dilution at 10 µM
L452R, E484K and E484Q substitutions	PCR2.F	*Primer Forward 1283U21*	ATTTTACAGGCTGCGTTATAG	0.5 µL
	PCR2.R	*Primer Reverse 1548L19*	TGCTGGTGCATGTAGAAGT	0.5 µL
	IC2.P	*Probe IC2 SARS-CoV-2*	JOE–TACCRGCCTGATAGATTTCAGTTG–BHQ1	0.25 µL
	452R.P	*Probe SARS-CoV-2 L452R*	FAM–CCGGTATAGATTGTTTAGGA–MGB	0.25 µL
	484K.P	*Probe SARS-CoV-2 E484K*	Cy5–CCTTGTAATGGTGTTAAAGGT–MGB	0.25 µL
	484Q.P	*Probe SARS-CoV-2 E484Q*	ROX–CTTGTAATGGTGTTCAAGGT–MGB	0.25 µL

**Table 2:** Analytical and clinical performance of the two variant-screening RT-PCRs.

		**Prevalence**	**Analytical Performance**		**Clinical Performance**			
			Sensitivity	Specificity	Sensitivity	Specificity	Positive Predictive Value	Negative Predictive Value
**RT-PCR#1**	69-70del	53.9%	500 IU/mL	100.0%	99.4%	100.0%	100.0%	99.3%
	N501Y	75.1%	500 IU/mL		100.0%	100.0%	100.0%	100.0%
**RT-PCR#2**	E484K	85.7%	1000 IU/mL	100.0%	92.9%	99.9%	92.9%	99.9%
	E484Q	1.5%	2000 IU/mL		100.0%	100.0%	100.0%	100.0%
	L452R	1.5%	2000 IU/mL		99.9%	96.2%	99.4%	99.2%

**Table 3:** Variant-screening RT-PCR#1 interpretation.

**Amplification**			**Interpretation**	**Suspected Variants**	
FAM	JOE	Cy5		First period of use [February–May 2021]	Second period of use [December 2021–February 2022]
(IC)	(69–70del)	(N501Y)			
+ *	+	+	No mutation detected	No Alpha, Beta or Gamma variant	Delta
+	−	−	69–70 deletion and N501Y substitution detected	Alpha	Omicron BA.1
+	+	−	N501Y substitution detected	Beta or Gamma	Omicron BA.2 or Deltacron XD or B.1.640 variant
+	−	+	69–70 deletion detected	No Alpha, Beta or Gamma variant	No Delta or Omicron variant
− **	−	−	Insufficient amount of virus or PCR inhibition	Noncontributive result requiring NGS ***	Noncontributive result requiring NGS

* Presence of an amplification curve, ** absence of amplification curve, *** next-generation sequencing.

**Table 4:** Comparison of variant-screening RT-PCRs’ results and NGS data.

RT-PCR#1 Amplification				NGS Results		RT-PCR#2 Amplification					NGS Results	
Sample ID	Ct FAM	Ct JOE	Ct Cy5	Clade/Lineage	Deletion/Substitution Detected	Sample ID	Ct JOE	Ct FAM	Ct ROX	Ct Cy5	Clade/Lineage	Substitution Detected
	(IC)	(69–70 del)	(N501Y)				(IC)	(L452R)	(E484Q)	(E484K)		
**1**	Pos (Ct < 37)	Neg	Neg	20I (Alpha, V1)	69–70del + N501Y	**31**	Pos (Ct < 37)	Pos	Neg	Neg	19B/501Y *	L452R
**2**	Pos (Ct < 37)	Neg	Neg	20I (Alpha, V1)	69–70del + N501Y	**32**	Pos (Ct < 37)	Neg	Neg	Neg	20A_B.1.640 *	None
**3**	Pos (Ct < 37)	Neg	Neg	20I (Alpha, V1)	69–70del + N501Y	**33**	Pos (Ct < 37)	Neg	Neg	Pos	20B/681H *	E484K
**4**	Pos (Ct < 37)	Neg	Neg	20I (Alpha, V1)	69–70del + N501Y	**34**	Pos (Ct < 37)	Pos	Neg	Neg	20D *	L452R
**5**	Pos (Ct < 37)	Neg	Neg	20I (Alpha, V1)	69–70del + N501Y	**35**	Pos (Ct < 37)	Neg	Neg	Pos	20H (Beta, V2)	E484K
**6**	Pos (Ct < 37)	Neg	Neg	20I (Alpha, V1)	69–70del + N501Y	**36**	Pos (Ct < 37)	Neg	Neg	Pos	20H (Beta, V2)	E484K
**7**	Pos (Ct < 37)	Neg	Neg	20I (Alpha, V1)	69–70del + N501Y	**37**	Pos (Ct < 37)	Neg	Neg	Pos	20H (Beta, V2)	E484K
**8**	Pos (Ct < 37)	Pos	Neg	20H (Beta, V2)	N501Y	**38**	Pos (Ct < 37)	Neg	Neg	Pos	20H (Beta, V2)	E484K
**9**	Pos (Ct < 37)	Pos	Neg	20H (Beta, V2)	N501Y	**39**	Pos (Ct < 37)	Neg	Neg	Pos	20H (Beta, V2)	E484K
**10**	Pos (Ct < 37)	Pos	Neg	20H (Beta, V2)	N501Y	**40**	Pos (Ct < 37)	Neg	Neg	Pos	20I (Alpha, V1)	E484K
**11**	Pos (Ct < 37)	Pos	Neg	20H (Beta, V2)	N501Y	**41**	Pos (Ct < 37)	Neg	Neg	Pos	20I (Alpha, V1)	E484K
**12**	Pos (Ct < 37)	Pos	Neg	20H (Beta, V2)	N501Y	**42**	Pos (Ct < 37)	Neg	Neg	Pos	20J (Gamma, V3)	E484K
**13**	Pos (Ct < 37)	Pos	Neg	20H (Beta, V2)	N501Y	**43**	Pos (Ct < 37)	Pos	Neg	Neg	21A (Delta)	L452R
**14**	Pos (Ct < 37)	Pos	Neg	20H (Beta, V2)	N501Y	**44**	Pos (Ct < 37)	Pos	Neg	Neg	21A (Delta)	L452R
**15**	Pos (Ct < 37)	Pos	Neg	20J (Gamma, V3)	N501Y	**45**	Pos (Ct < 37)	Pos	Neg	Neg	21A (Delta)	L452R
**16**	Pos (Ct < 37)	Pos	Neg	20J (Gamma, V3)	N501Y	**46**	Pos (Ct < 37)	Pos	Neg	Neg	21A (Delta)	L452R
**17**	Pos (Ct < 37)	Pos	Neg	20J (Gamma, V3)	N501Y	**47**	Pos (Ct < 37)	Pos	Neg	Neg	21A (Delta)	L452R
**18**	Pos (Ct < 37)	Neg	Neg	21K (Omicron, BA.1)	69–70del + N501Y	**48**	Pos (Ct < 37)	Neg	Neg	Pos	21H (Mu) *	E484K
**19**	Pos (Ct < 37)	Neg	Neg	21K (Omicron, BA.1)	69–70del + N501Y	**49**	Pos (Ct < 37)	Pos	Neg	Neg	21I (Delta)	L452R
**20**	Pos (Ct < 37)	Neg	Neg	21K (Omicron, BA.1)	69–70del + N501Y	**50**	Pos (Ct < 37)	Pos	Pos	Neg	21J (Delta)	L452R +E484Q
**21**	Pos (Ct < 37)	Neg	Neg	21K (Omicron, BA.1)	69–70del + N501Y	**51**	Pos (Ct < 37)	Pos	Pos	Neg	21J (Delta)	L452R +E484Q
**22**	Pos (Ct < 37)	Neg	Neg	21K (Omicron, BA.1)	69–70del + N501Y	**52**	Pos (Ct < 37)	Pos	Pos	Neg	21J (Delta)	L452R +E484Q
**23**	Pos (Ct < 37)	Neg	Neg	21K (Omicron, BA.1)	69–70del + N501Y	**53**	Pos (Ct < 37)	Pos	Pos	Neg	21J (Delta)	L452R +E484Q
**24**	Pos (Ct < 37)	Pos	Neg	21L (Omicron, BA.2)	N501Y	**54**	Pos (Ct < 37)	Pos	Pos	Neg	21J (Delta)	L452R +E484Q
**25**	Pos (Ct < 37)	Pos	Neg	21L (Omicron, BA.2)	N501Y	**55**	Pos (Ct < 37)	Pos	Pos	Neg	21J (Delta)	L452R +E484Q
**26**	Pos (Ct < 37)	Pos	Neg	21L (Omicron, BA.2)	N501Y	**56**	Pos (Ct < 37)	Pos	Pos	Neg	21J (Delta)	L452R +E484Q
**27**	Pos (Ct < 37)	Pos	Neg	21L (Omicron, BA.2)	N501Y	**57**	Pos (Ct < 37)	Pos	Pos	Neg	21J (Delta)	L452R +E484Q
**28**	Pos (Ct < 37)	Pos	Neg	21L (Omicron, BA.2)	N501Y	**58**	Pos (Ct < 37)	Pos	Pos	Neg	21J (Delta)	L452R +E484Q
**29**	Pos (Ct < 37)	Pos	Pos	21J (Delta)	None	**59**	Pos (Ct < 37)	Neg	Neg	Neg	21K (Omicron, BA.1)	None
**30**	Pos (Ct < 37)	Pos	Pos	21J (Delta)	None	**60**	Pos (Ct < 37)	Neg	Neg	Neg	21K (Omicron, BA.1)	None

Ct = cycle threshold, IC = internal control, Pos = presence of an amplification curve, Neg = no amplification, NGS = next-generation sequencing, * clades/lineages corresponding to VOIs (variants of interest).

**Table 5:** Ct values of the linearity and efficiency evaluation, for the two variant-screening RT-PCRs.

		RT-PCR#1									RT-PCR#2		
WHO–IS SCV-2 RNA Concentration		Ct FAM (IC)			Ct Cy5 (N501Y)			Ct JOE (69–70 del)			Ct JOE (IC)		
IU/mL	Log_10_ IU/mL	Replicate 1	Replicate 2	Mean value	Replicate 1	Replicate 2	Mean value	Replicate 1	Replicate 2	Mean value	Replicate 1	Replicate 2	Mean value
5,011,872	6.7	24.29	23.92	24.11	25.99	26.26	26.13	23.92	23.89	23.91	25.44	26.06	25.75
501,187	5.7	27.07	27.42	27.25	28.8	29.07	28.94	26.52	27.07	26.80	28.45	29.38	28.92
50,119	4.7	31.19	31.08	31.14	32.85	32.59	32.72	30.58	30.51	30.55	32.96	32.73	32.85
5012	3.7	35.5	35.91	35.71	36.09	37.55	36.82	34.95	34.47	34.71	37.3	36.96	37.13
501	2.7	38.66	37.5	38.08	39.36	40.17	39.77	38.18	37.04	37.61	41.35	41.39	41.37
250	2.4	Neg	Neg		40.35	41.16	40.76	Neg	Neg		Neg	Neg	
125	2.1	Neg	Neg		Neg	43.14		Neg	Neg		Neg	Neg	
62.5	1.8	Neg	Neg		Neg	Neg		Neg	Neg		Neg	Neg	

The dotted line corresponds to the limit of detection. IU = international unit, Ct = cycle threshold, IC = internal control, Neg = absence of amplification.

**Table 6:** Variant-screening RT-PCR#2 interpretation.

Amplification				Interpretation	Suspected Variants
JOE	FAM	Cy5	ROX		
(IC)	(L452R)	(E484K)	(E484Q)		
+ *	−	−	−	No mutation detected	Circulating VOIs
+	+	−	−	L452R substitution detected	Delta
+	−	+	−	E484K substitution detected	Beta or Gamma
+	−	−	+	E484Q substitution detected	Noncontributive result requiring NGS ***
+	+	+	−	L452R and E484K substitutions detected	Noncontributive result requiring NGS
− **	−	−	−	Insufficient amount of virus or PCR inhibition	Noncontributive result requiring NGS

* Presence of an amplification curve, ** absence of amplification curve, *** next-generation sequencing.

## Data Availability

Not applicable.
